# A Morbid View of the over-65's
*A lecture to the Bristol Branch of the British Medical Association.


**Published:** 1956-04

**Authors:** G. Stewart Smith

**Affiliations:** Area Pathologist, Exeter


### A MORBID VIEW OF THE OVER-65's*

BY

G. STEWART SMITH, M.A., M.D.

*Area Pathologist, Exeter*

In this rather cryptic title morbid means through the eyes of a morbid anatomist. I thought it might be opportune for a pathologist to add his views to those of the clinicians and sociologists on some of the problems created by this age group. It happens that my own autopsy experience has given me an unusually high proportion of elderly subjects. Some five years ago I analysed a group of some 1,700 autopsies in subjects over 65 years of age collected from the Manchester Municipal Hospitals, which tended, at that time at least, to have a high proportion of older patients and also to be receptacles of lost causes of all sorts. Since I came to Exeter in 1949, I have, for a different reason, still to study many elderly people and I find that more than a third of all my autopsies are on people more than 65 years of age.

My cases are nearly all from one busy general hospital, the Royal Devon and Exeter, with a proportion of sudden and unexplained deaths investigated for the Coroner. I assume therefore that the age distribution reflects the contentment and peace of Devon.

This lecture then is based on an analysis of the causes of death of 669 people, aged 65 years and over, collected during the last five years. I am fully aware that mortality is a very different matter from morbidity: a great deal has been said about morbidity in these older people; perhaps less has been said about the actual pathological findings.

There are several reasons why the age of 65 should be chosen as a dividing line. Two may be noted:

(1) It is still the normal retiring age for most poeple and the time when many of us hope to be eligible for pensions: certainly it is a time when we tend to slack off a little from our normal routine. I should however point out that Sheldon (1954) puts the dividing line at 70–75. He says that before 70 the majority are potential donors to the common effort and after 70 they are potential debtors.

(2) It is the age which is used in the Registrar-General's statistics. Let us not forget however how much our ideas have changed. Not many years ago I was designing punch cards for tumour specimens and was considering the age groupings. My young assistant said, " You can lump all those over 60 into one group ", indicating that in his opinion all people over 60 belonged to the limbo lost. Thirty years ago when I was doing an H.P. job I was quite prepared to sign up anyone over 65 as "senility" and some at 60 as " premature senility ".

Circumstances now compel us to take a different view of these things. It is common knowledge that we are an ageing population since the expectation of life is greater than ever before. Table I, the Registrar-General's Statistical Review of 1950 and Table II, from the recent Report of the Phillips Committee (1954), show this quite clearly. To put this matter in another and very simple form—100 years ago the number of elderly people in Great Britain was 3 per cent. of the population, now it is 11 per cent. and by the end of the century it will propbably be 20 per cent. If we are to get the best out of the older age group we must study them and see what special medical and surgical problems they present.

* A lecture to the Bristol Branch of the British Medical Association.

TABLE I

EXPECTATION IN YEARS

| AGE. | MALES. | FEMALES. |
|---|---|---|
| 65 | 12·00. | 14·39. |
| 70 | 9·31. | 11·07. |
| 75 | 7·03. | 8·26. |
| 80 | 5·24. | 6·11. |
| 85 | 3·95 | 4·56. |

TABLE II

POPULATION OF PERSONS IN ENGLAND AND WALES BY AGES, PER 10,000 AT ALL AGES, 1901, 1911, 1921, 1931, 1939, 1951

| AGE. | 1901. CENSUS. | 1911 CENSUS. | 1921 CENSUS. | 1931 CENSUS. | 1939 MID-YEAR. | 1951. CENSUS. |
|---|---|---|---|---|---|---|
| 65 | 331 | 377 | 434 | 536 | 643 | 737 |
| 75 | 121 | 126 | 151 | 182 | 225 | 306 |
| 85 AND OVER | 15 | 18 | 20 | 24 | 29 | 43 |

What sources of information are available for study? (1) The Registrar-General's figures (2) Autopsy observations, including one's own.

The Registrar-General's figures are the only one's available for the study of the population as a whole and it must be remembered that he deals with some 500,000 deaths per year. How near his figures approximate to the truth I don't know but he himself admits three difficulties:

(1) The quality of diagnostic services during life varies much in different parts of the Country. Perhaps in time the National Health Service will even this out a bit.

(2) Only a proportion of the causes certified are confirmed by autopsy. (I am more convinced than ever that it is seldom that autopsy fails either to modify or to change the diagnosis.)

(3) The real cause of death may not be stated on the certificate because it is unpleasant or is inconvenient to the relatives.

When a pathologist discusses his own experience problems also arise. The series studied must be large and consecutive and no stress must be laid on small differences. All hospital cases are to a varying degree selected and it is very difficult to say how far the results obtained apply generally to the area concerned. Another problem is how to analyse the results: this is not easy and there is bound to be some overlapping but perhaps it can be accepted as roughly accurate. It is interesting to note that in no case has the cause of death been returned purely as senility. Warthin, writing in 1928, stated that in 35 years of morbid anatomy he had only had 25 autopsies in which biological death could be presumed. The classifications found in the Registrar-General's tables have also moved away from the diagnosis of senility to something more specific and I would like to think that it is a distinction with a difference. (Table III). In one place the Registrar-General makes the suggestion that his groupings may not be exact but are probably near the truth and I think this is probably so, especially with respect to cardiovascular disease, which in figures for the Country and in my own local survey makes up about two-fifths of the causes of death.

TABLE III

REGISTRAR GENERAL'S FIGURES FOR 5 PRINCIPAL CAUSES OF DEATH AGE 65 AND OVER (PER MILLION LIVING)

| | MALES. | | FEMALES. | |
|---|---|---|---|---|
| 1848 TO 1872 | OLD AGE. | 27,726. | OLD AGE. | 30,695. |
| | BRONCHITIS. | 11,007. | BRONCHITIS. | 10,487. |
| | HEART DISEASES. | 6,572. | PARALYSIS. | 5,858. |
| | PARALYSIS. | 6,153. | HEART DISEASES. | 5,811. |
| | APOPLEXY. | 4,486. | DROPSY. | 4,045. |
| 1901 TO 1910 | OLD AGE. | 18,339. | OLD AGE. | 18,768. |
| | HEART DISEASES. | 13,329. | HEART DISEASES. | 12,059. |
| | BRONCHITIS. | 10,399. | BRONCHITIS. | 10,441. |
| | CANCER. | 6,997. | CANCER. | 7,023. |
| | CEREBRAL HAEM: EMBOLISM. | 5,298. | CEREBRAL HAEM: | 5,097. |
| 1950 | HEART DISEASES. | 26,815. | HEART DISEASES. | 21,766 |
| | CANCER. | 12,009. | VASCULAR LESIONS AFFECTING C.N.S. | 10,922. |
| | VASCULAR LESIONS AFFECTING C.N.S. | 10,784. | CANCER. | 8,273. |
| | BRONCHITIS. | 5,853. | BRONCHITIS. | 3,160. |
| | HYPERTENSIVE DISEASE. | 3,460. | HYPERTENSIVE DISEASE. | 2,901. |

For the purpose of this paper I have divided these 669 autopsies into 12 groups and have made a distinction between the age groups 65–75 and over 75 and between males and females, (Table IV).

One would expect the first three groups—coronary disease, general arteriosclerosis and hypertension and cancer—to account for more than one-half of the deaths. Page (1945) stated that " The problem of arteriosclerosis is the hub of the problem of cardio-vascular disease. Notwithstanding its great importance our knowledge is sketchy regarding its aetiology, indefinite as to classification and meagre as to prevention and cure." As far as I know this is still true.

Let us now consider these groups and see if they have anything to teach us.

TABLE IV

SUMMARY OF 669 AUTOPSIES AGED 65 AND OVER (EXETER)

| | FEMALES. | | | MALES. | | | TOTAL. |
|---|---|---|---|---|---|---|---|
| | 65-74 | OVER 75 | BOTH GROUPS. | 65-74 | OVER 75 | BOTH GROUPS. | |
| CORONARY. | 38 | 23 | 61 | 60 | 28 | 88 | 149. |
| ARTERIOSCLEROSIS. | 29 | 19 | 48 | 24 | 22 | 46 | 94. |
| NEOPLASM. | 36 | 17 | 53 | 66 | 15 | 81 | 134. |
| CEREBRAL HAEM: AND THROMBOSIS. | 8 | 4 | 12 | 11 | - | 11 | 23. |
| INFECTION. | 9 | 4 | 13 | 13 | 8 | 21 | 34. |
| PROSTATE AND KIDNEY. | 7 | 2 | 9 | 31 | 14 | 45 | 54 |
| PULMONARY EMBOLISM. | 5 | 1 | 6 | 4 | - | 4 | 10. |
| ABDOMINAL CRISES. | 17 | 9 | 26 | 34 | 25 | 59 | 85. |
| POISONING. | 7 | 3 | 10 | 3 | 6 | 9 | 19. |
| BRONCHITIS. | 4 | 1 | 5 | 6 | - | 6 | 11. |
| TRAUMA. | 15 | 8 | 23 | 12 | 13 | 25 | 48. |
| MISCELLANEOUS. | 3 | - | 3 | 3 | 2 | 5 | 8. |

GROUP I. CORONARY ARTERY DISEASE

This accounts for about a quarter of the deaths and a high proportion of the sudden deaths. In this series there is very little difference between the sexes. In the younger age groups there is certainly a predominance of males but after 65 the incidence has evened out in rather striking fashion. Further analysis shows that the type of lesion was similar in men and women. There was evidence of old ischaemia, either patches of fibrosis or a large scar representing a healed infarction in about half the cases while in the other half it seemed, judging from naked-eye appearances, that the first attack had been fatal. In half of this group, i.e. in a quarter of the whole coronary group, there had been time for a fully developed infarct. The left coronary, and especially the anterior descending branch, was much more commonly the site of the fatal occlusion than the right.

Three points have struck me on the clinical side:—(1) The fact that some practitioners are still not aware of the frequency of this condition in older people and do not consider it as one of the very likely diagnoses in a patient complaining, perhaps for the first time, of vague digestive trouble and epigastric pain, which are often the first signs. (2) The electrocardiagram, regarded in some quarters as almost infallible because it writes down its results, can often lead one astray. A positive reading, indicating myocardial ischaemia or infarction, has to be taken seriously but a negative finding does not necessarily exclude gross infarction. (3) The first attack, as represented by a large fully healed infarct, quite often seems to have been almost silent or perhaps accompanied or followed by some breathlessness on exertion and no actual pain.

Coronary disease undoubtedly remains one of the big bugbears which prevents people who have survived all the other hazards, some of which have diminished so strikingly during the last 10 or 15 years, from dying a biological death. I think we must realize that coronary occlusion in this age group is almost entirely a problem of arteriosclerosis in general. Mortality from heart disease must eventually depend on either insufficiency of the coronary circulation or disease of the myocardium and in fact about two-thirds of all heart disease and heart failure arise from relative or absolute failure of the coronary vascular tree. The whole problem both from the experimental and from the human side has recently been reviewed in a monograph by Gregg (1945), " The coronary circulation in health and disease ", and it is worth while seeing what he has to say. He states that occlusion of the coronaries may arise from a number of events associated with the arteriosclerotic process: (1) fragments of atheromatous plaques may rarely break off and obstruct the lumen lower down (2) the plaques themselves may become so large as to obstruct the lumen (3) thrombosis may occur (4) arteriosclerotic lesions may become richly vascularized and intramural haematoma may arise from rupture of one of these. This occurrence may be an important cause of trouble in hypertensives and looking back on some of my cases I think may well have happened, especially when the lumen is found to be obstructed by much old yellowish-brown material.

In spite of all the experimental work summarized so well by Gregg the aetiological factor is not clear and there are many seemingly contractictory pieces of evidence. Atheromatous lesions can certainly be produced in rabbits by feeding them for long periods with large amounts of cholesterol, a substance alien to their usual diet, and in dogs by giving them cholesterol and thiouracil, but in man arteriosclerosis and coronary disease are not usually accompanied by hypercholesterolaemia. On the other hand, conditions in which there is an increase of cholesterol in the blood, such as diabetes, myxoedema and xanthomatosis, do have a high incidence of arteriosclerosis and often develop it at an early age.

Several surveys have shown an increase of coronary disease in men who are overweight but this cannot account for the general lower incidence in women. Great importance has been attached to the fact that there is a low incidence of general and coronary atherosclerosis in China and Costa Rica where nutrition is poor and where apparently placid and philosophical dispositions are, or were, the rule! I was much impressed during a recent visit to America with some papers on the demographic pathology of cancer. Pathologists from all parts of the world gave their experiences of the distribution of cancer among their populations and I think that in time this may give very valuable information. It might be that a demographic survey of arteriosclerosis would be helpful.

There is no doubt that the condition in this older group is different in some ways from that found in younger people where there is often no generalized arteriosclerosis and the whole fatal lesion is confined to one or two centimetres of the left coronary artery or one of its main branches. Experimental work has shown quite clearly that anastomoses, at the arteriolar level, do exist in the coronary system and can dilate up to ten times their normal size and also, and this is important, that the process goes on over days and weeks. This gives a logical reason why adequate rest is required after the first attack and why in this older group where the occluding process may develop slowly the heart can still carry on with grossly diseased coronaries.

In looking at my cases I have often wondered what place anti-coagulants really have. Recent thrombosis in the coronaries themselves does not seem to be a major factor in most of them or at least is only the terminal event. I am not a clinician and am confused by the diametrically opposed views of the experts. Gilchrist and Tullock (1954) say that the objects of anti-coagulant therapy are (1) to prevent any extension or propagation of the original thrombus if present (2) to prevent secondary thrombi, either endocardial overlying the infarct or in the peripheral veins of the legs (3) to promote dissolution, absorption and re-canalisation of the original or secondary thrombi. They consider that anti-coagulants are capable of halving the death rate.

Opposed to this is the view of Russek and Zohman (1954) who, as a result of their work in the U.S.P.H.S. Cardiovascular Research Unit, say that anti-coagulants are neither necessary nor desirable for patients having their first attack and presenting no unfavourable criteria for recovery at the time of their first examination. Only about 30 per cent. of all patients with acute myocardial infarction need anti-coagulants and age is of no significance either for the immediate prognosis or as an indication or contra-indication for anti-coagulants. All should depend on the patient's initial appearance.

I think I can say that in some of my cases the occurrence of femoral venous thrombosis and pulmonary embolism might have been prevented by anti-coagulants: seldom do I think they would have influenced the primary heart lesion and I am not aware that embolism from the mural clot over an infarct has been responsible for death in many cases.

Just recently Wright, Marple and Beck (1955) have published in America a study of 1,031 cases of coronary disease. They mention that in the U.S.A. every year 200,000 persons die from myocardial infarction and 600,000 to 800,000 suffer attacks; 52 per cent. of the patients were under 60 years of age and of these 87·3 per cent. were males. About half were given anti-coagulants and half were not, and putting the matter very briefly they conclude that the benefit of anti-coagulants was apparent in all age groups but greatest in the 50–69 group, that anti-coagulants are effective in both mild and severe cases and that both types will benefit by protection from the outset. They suggest that the screening for good-risk cases involves a great possibility of error.

Faced with divergent views and not being a clinician I asked an opinion from a cardiologist friend who summed up the position as follows: There are three groups of cases: (1) those with a mild lesion where pain is of brief duration, illness short and patients active in bed from an early stage with very little fall of blood pressure; (3) those with profound shock and great fall in blood pressure who are obviously not going to do well. These two groups do not require anti-coagulants. (2) The intermediate group where the decision is a problem. In this group, while anti-coagulants may have no effect whatever on the coronary arteries they may prevent the formation of mural clots and tend to diminish the liability to venous thrombosis in the legs or pelvis.

My own feeling is that owing to the more generalized atheroma in the older age groups anti-coagulants are likely to be less effective than in younger people but I entirely agree that if they are given at all they must be given in a dosage which keeps the prothrombin time about 2½ times the normal and should be continued for some weeks. This necessitates adequate laboratory control. One does see examples of hesitant and inadequate treatment which can only be helpful to the doctor's mind and not of much use to the patient.

### GROUP 2. ARTERIOSCLEROSIS

This is composed of cases where the cause of death was thought to be due to general arterial degeneration and hypertension not specifically or chiefly affecting the coronary arteries. Much of what I have said about coronary arteriosclerosis applies here, including the fact that we have not found the answer to this problem. I think it is accepted however that the physiological changes in arteries due to ageing (deterioration of the elastic tissue and dilatation of the lumen) are not synomymous with arteriosclerosis which is patchy and affects the intima chiefly. It is of course well known that atheroma can start almost from birth. Still I suppose this group does represent the nearest approach to what 30 years ago would have been called senility. About one-seventh of the deaths were in this group and they include several dissecting aneurysms.

The surprising thing very often is not that the patients have died but how long they have lived, and in many cases been reasonably active, with such gross and generalized changes; the fact that the development of the condition is so gradual is I suppose the answer as it gives the body time to work up every sort of device to ensure an adequate circulation. As long as the blood supply to the heart itself is reasonable the rest of the body can put up with an awful lot.

Another point which interests me, and to which I do not know the real answer, is how rarely peripheral emboli causing gangrene occur even when the aorta shows pronounced ulceration and is covered with large masses of blood clot.

Haemopericardium, in which the pericardial sac contained a mass of blood clot occurred in connexion with Groups 1 and 2 twenty-three times. I have seen so many of these in the South-West that I am no longer excited by them but they often give rise to sudden and dramatic death. In fifteen of these the haemorrhage was due to a ruptured acute infarct and in eight due to intra-pericardial extension and rupture of a dissecting aneurysm.

### GROUP 3. NEOPLASMS

This group contains 134 cases, about one-fifth of the total cases under review, and constitutes the second largest cause of death as one would expect (79 were males and 55 females). I have included in this group cases in which death was accelerated by surgical intervention, some surgeons now disliking the term "interference". Even so, this figure does not I am sure represent anything like the total number dying from this condition as many cases, clearly verified by biopsy or radiography or by gross clinical findings, are not subjected to autopsy and many die at home.

Willis in his book on tumours suggests that the mortality figures for cancer, taken from death certificates, may not be far short of the "real" total but that figures for individual sites may have a wide margin of error, especially for internal cancer. This I think may well be true. Table V shows the sites of election in my series—lung, colon, stomach, rectum and prostate in men and pancreas, colon, stomach, rectum, uterus and gall-bladder in women. I cannot lay a stress on the lower incidence of lung cancer as compared with my Manchester series: there I was dealing with municipal hospitals where so many of these cases used to end their days.

I have only one general observation to make on this group—the condition was allowed to get to a surprisingly obvious stage in some of them before the patient, or perhaps occasionally the doctor, tumbled to the diagnosis.

### GROUP 4. CEREBRAL HAEMORRHAGE AND THROMBOSIS

This group contains only 23 cases and seems a rather less common cause of death than one would expect.

TABLE V

NEOPLASMS
TOTAL CASES 134

| SITE. | MALE. | FEMALE. |
|---|---|---|
| LUNG. | 11 | 2 |
| STOMACH. | 8 | 5 |
| COLON. | 9 | 5 |
| RECTUM. | 11 | 4 |
| OESOPHAGUS. | 6 | 2 |
| GALL BLADDER AND DUCTS. | 1 | 4 |
| PANCREAS. | 1 | 4 |
| KIDNEY. | 6 | 1 |
| PROSTATE. | 7 | – |
| BLADDER. | 4 | 2 |
| UTERUS (BODY). | – | 4 |
| UTERUS (CERVIX). | – | 2 |
| OVARY. | – | 3 |
| THYROID. | 1 | 2 |
| GLIOBLASTOMA. | 2 | 1 |

GROUP 5. PULMONARY EMBOLISM

This was found on 46 occasions; as a "primary" condition following thrombosis of the veins of the legs in 10 cases but in 36 others, of equal sex distribution, as a complication or terminal event. It generally followed infection, trauma or operation or was secondary to heart failure. Although I am limiting my remarks to the over-65 group I see quite a lot of fatal pulmonary embolism in lower age groups and it is undoubtedly an important condition. Three things have struck me in my personal series: (1) Not all housemen are as aware as they should be of the frequency of this condition and sometimes inspection of the legs in patients likely to develop it is neglected. Swelling of one leg has frequently been noted for the first time on the autopsy table. (2) Some of the thromboses in the deeper leg veins seem to occur without either swelling or the classical pain and tenderness in the calf when patients are lying in bed. (3) In most cases the massive fatal embolism in which many inches of clot come away is not accompanied by evidence of preceding smaller emboli.

This subject has been discussed in a leading article in *The Lancet* (1955) in which it is called the "acute disease most likely to escape detection" and it is emphasized that the recognition of pulmonary embolism depends on constant vigilance. Deep-vein thrombosis should be treated immediately by anti-coagulants since in 10 per cent. of patients it is followed by fatal pulmonary embolism; treatment after a single small embolism may reduce the likelihood of a recurrence. Some authors go so far as to suggest routine anti-coagulant therapy during the danger period when the patient first gets out of bed after operation or a serious medical condition. It is pointed out that pulmonary embolism is likely to grow in importance as a cause of death as the population ages.

### GROUP 6. INFECTIONS

These present a wide scatter of causes and comprise only 34 cases (5 per cent. of the total). The influence of antibiotics and sulphonamides is seen very dramatically here. There were only nine deaths from lobar pneumonia, which used to be described as the " Captain of the men of death ", and a similar number from broncho-pneumonia. I well remember from my own experience when lobar pneumonia in this age group had a mortality between 25 and 30 per cent. In my previous larger series of cases from Manchester (Table VI) infections caused about 11 per cent. of the deaths. The present figures probably represent the lowest reasonable figure obtainable.

TABLE VI

SUMMARY OF 1677 AUTOPSIES ON PATIENTS 65 YEARS OF AGE AND OVER (MANCHESTER SERIES)

| CAUSE OF DEATH. | MALE. | FEMALE. | TOTAL. | PERCENTAGE. |
|---|---|---|---|---|
| CORONARY OCCLUSION. | 58 | 45 | 103 | 5·7 |
| ATHEROSCLEROSIS AND HYPERTENSION. | 90 | 70 | 160 | 9·0 |
| CEREBRAL HAEMORRHAGE AND THROMBOSIS. | 48 | 44 | 92 | 5·0 |
| CHRONIC BRONCHITIS AND BRONCHIECTASIS. | 76 | 28 | 104 | 5·8 |
| PROSTATE. | 144 | — | 144 | 8·0 |
| ACUTE INFECTIONS. | 90 | 54 | 144 | 8·0 |
| ACUTE ABDOMEN. | 73 | 86 | 159 | 9·0 |
| GASTRIC AND DUODENAL ULCERS | 66 | 27 | 93 | 5·2 |
| CHRONIC NEPHRITIS. | 14 | 17 | 31 | 1·7 |
| BLOOD DISEASES. | 16 | 18 | 34 | 1·9 |
| TRAUMA. | 24 | 24 | 48 | 2·7 |
| TUBERCULOSIS. | 41 | 12 | 53 | 3·0 |
| CANCER. | 342 | 170 | 512 | 28·5 |
| TOTALS. | 1082 | 595 | 1677 | |

### GROUP 7. RENAL LESIONS

The 54 cases in this group were mostly men and essentially mean deaths due to enlargement of the prostate and its consequences—cystitis, pyelonephritis, uraemia and post-operative deaths. Many patients still leave it desperately late before they seek medical aid and education is badly needed. I found a similar lot of late and neglected cases in my earlier survey when operation was performed when the kidneys were bankrupt. A genito-urinary surgeon with whom I discussed this recently said there were some medical men who still thought the indications for surgery were either acute retention or overflow incontinence. Until we discover the hormonal cause of this condition and the antidote I imagine that earlier surgery would reduce the mortality.

### GROUP 8. ABDOMINAL CRISES

I think the group of abdominal conditions warrants further consideration as it makes up some 8 per cent. of the total. The distribution is shown in Table VII. It is of course common experience that for one reason or another some of these cases seek treatment at a very late stage and some will always represent bad risks.

TABLE VII

ABDOMINAL CRISES
TOTAL CASES 85

| | MALE. | FEMALE. |
|---|---|---|
| HERNIA. | 12 | 2 |
| CHOLECYSTITIS. | 4 | 5 |
| PERITONEAL ADHESIONS. | 7 | 2 |
| PERFORATED GASTRIC ULCER. | 5 | 4 |
| GASTRIC ULCER – HAEMORRHAGE. | 8 | 1 |
| PERFORATED DUODENAL ULCER. | 5 | 0 |
| DUODENAL ULCER – HAEMORRHAGE. | 3 | 3 |
| HAEMORRHAGIC PANCREATITIS. | 2 | 4 |
| DIVERTICULITIS. | 5 | 4 |
| ACUTE APPENDIX. | 2 | 0 |

A development which has interested me, especially since working in the South-West, is that age alone does not now contra-indicate quite extensive surgery and with modern anaesthesia and antibiotics a surgeon can assess a condition on its merits without worrying too much about the age factor. The outlook in this respect has undoubtedly changed for the better.

### GROUP 9. BRONCHITIS AND BRONCHIECTASIS

Bronchitis is not an important cause of death in my series, even in the over-75 group. It is considerably less than in the Manchester series due partly to climatic conditions and partly to the fact that antibiotics ward off or cure pneumonia and other chest infections which used to lead to a fatal termination in many of these people.

GROUPS 10 AND 11

Poisoning (I have maliciously included one or two anaesthetic deaths in this group) and trauma do not need further discussion.

When the Exeter figures are compared with those obtained from an earlier survey of some 1,700 autopsies in the same age group from the Manchester Municipal Hospitals (Table VI) two points must be borne in mind; (1) the lower figures for infections in the Exeter group are no doubt due to sulphonamides and antibiotics (2) the higher incidence of cancer in the Manchester figures is probably due to the fact that during the period 1938–48 a large number of cancer cases tended to end their days in municipal hospitals.

Perhaps I can sum up as follows:

(1) The three chief causes of death in this series were coronary occlusion, cancer and generalized arteriosclerosis in that order, and so far as these groups are concerned do not differ very widely from the Registrar-General's figures for the Country as a whole. The answer to these three problems we do not yet know. Cancer, as things are, will remain of increasing importance as the population ages.

(2) Infection has ceased to occupy the prominent place it once held.

(3) Chronic bronchitis does not appear to be one of the big five causes of death in older people living in and around Exeter.

(4) There is room for improvement in the deaths due to acute abdominal conditions and to enlargement of the prostate and its complications.

In conclusion I would like to say that I have only given a short review of one pathologist's work in one age group in a centre in the South-West and some *obiter dicta* which spring from a contemplation of these figures. I make no claim that these figures mean anything more than that.

### REFERENCES

Anti-coagulants for Myocardial Infarction (1955). *Lancet*, **1**, 32.
Anderson, W. A. D. (1948). *Pathology*, 1st Edition, p. 574. Kimpton, London.
Gavey, G. J. (1949). *Lancet*, **2**, 725.
Gilcrist, A. R., and Tulloch, J. A. (1954). *B.M.J.*, **2**, 720.
Gregg, D. E. (1950). *Coronary Circulation in Health and Disease*, Kimpton, London.
Page, I. H. (1940). *Ann. Int. Med.*, **14**, 1741.
*Report of Committee on Economic and Financial Problems of Provision for Old Age*, (1954). Cmd. 9333. H.M. Stationery Office, London.
Russek, H. I., and Zohman, B. L. (1954). *Lancet*, **2**, 910.
Sheldon, J. H. (1954). *Lancet*, **2**, 151.
Warthin, A. S. (1928). *Bull. N.Y. Acad. Med.*, **4**, 1006.
Willis, R. A. (1953). *Pathology of Tumours*, 2nd Edition, p. 78. Butterworth and Co., London.

